# Editorial: Exploration of highly active enzymes, performance enhancement and enzymatic processing techniques

**DOI:** 10.3389/fbioe.2022.1119604

**Published:** 2022-12-15

**Authors:** Yi Zhang, Mingming Zheng

**Affiliations:** Hubei Hongshan Laboratory, Hubei Key Laboratory of Lipid Chemistry and Nutrition, Key Laboratory of Oilseeds Processing, Ministry of Agriculture, Oil Crops Research Institute, Chinese Academy of Agricultural Sciences, Wuhan, China

**Keywords:** highly active enzyme, enzyme immobilization, synthetic biology (synbio), enzymatic processing, food additives

Enzymes have great industrial potential as biocatalysts, which have attracted much attention on their extensive applications. Derived from an organism or cell culture that catalyzes substrate conversions, enzymes are used for the synthesis of target products in a mild temperature, pH, substrate specificity under suitable reaction. Therefore, it is highly important to explore and improve the highly active enzymes for increased productivity. Besides, the enzyme-based processing technologies with the advantages of high efficiency, energy-saving, clean, and sustainability shed light on its huge potential in green technology. For instance, the enzymatical conversion of waste-based stocks into value-added products is becoming a hot Research Topic in environmental protection. In recent years, due to industrial upgrading, green production by enzymatic catalysis has received particular attention from both economic and environmental aspects. Increased developments of new enzymes and improvement of current enzymes have emerged and advances in catalytic field have ushered in a new era.

This Research Topic is to present readers a Research Topic of most up-to-date progress in enzymatic techniques. In total, six papers are published in this Research Topic, including 1 brief research report and 5 research articles. In the following, we just briefly highlight the published papers. Zhou et al. developed a novel Chondroitin AC lyase (ChSaseAC) to favor the production of low molecular weight chondroitin sulfate with high specific activity and improved storage stability. By chemical modification with betaine ionic liquids and genetic engineering methods, the catalytic performance of *enzymes* was effectively enhanced (Shen et al.; Xue et al.). Similarly, Wang et al. immobilized *Candida rugosa* lipase (CRL) onto a nanocomposite (Fe3O4-CS-DAC) and also enabled to optimize the stability as well as reusability of enzymes. Meanwhile, Stevens and Shi attempted to improve the activity and stability of the multicopper oxidase enzyme laccase by modifying the enzyme’s surface charges *via* acetylation, succinylation, cationization or neutralization. Finally, Li et al. provided a promising approach for diabetes detection using co-immobilized horseradish peroxidase (HRP) and glucose oxidase (GOx) on hybrid DNA nanoflowers (GOx-HRP@hDFs).

We hope this Research Topic will benefit both the scientific and industrial communities to track the state-of-the art in this enzymatic field, no matter whether the readers are at a beginner or professional level. Finally, we thank all authors, reviewers and the editorial staffs of Frontiers in Bioengineering and Biotechnology for their contributions, valuable comments, and tireless support to make the success of this Research Topic.

